# Direct oHSV Infection Induces DC Maturation and a Tumor Therapeutic Response

**DOI:** 10.3390/v17081134

**Published:** 2025-08-19

**Authors:** Doyeon Kim, Michael Kelly, Jack Hedberg, Alexia K. Martin, Ilse Hernandez-Aguirre, Yeaseul Kim, Lily R. Cain, Ravi Dhital, Kevin A. Cassady

**Affiliations:** 1 Center for Childhood Cancer Research, Abigail Wexner Research Institute at Nationwide Children’s Hospital, Columbus, OH 43205, USA; doyeon.kim@nationwidechildrens.org (D.K.); mchkelly92@gmail.com (M.K.); Jack.Hedberg@nationwidechildrens.org (J.H.); alexia.martin@nationwidechildrens.org (A.K.M.); ilse.hernandez-aguirre@nationwidechildrens.org (I.H.-A.); yeaseul.kim@nationwidechildrens.org (Y.K.); lily.cain@nationwidechildrens.org (L.R.C.); ravi.dhital@nationwidechildrens.org (R.D.); 2 College of Medicine, The Ohio State University, Columbus, OH 43220, USA; 3 Department of Pediatrics, The Ohio State University College of Medicine, Columbus, OH 43220, USA; 4 Department of Pediatrics, Division of Pediatric Infectious Diseases, Nationwide Children’s Hospital, Columbus, OH 43205, USA

**Keywords:** dendritic cell (DC), oncolytic herpes simplex virus (oHSV), T cell priming, central nervous system (CNS) malignant tumors

## Abstract

Oncolytic herpes simplex virus (oHSV) is a promising cancer immunotherapy that induces tumor cell lysis and stimulates anti-tumor immunity. Our previous single-cell RNA sequencing analysis of oHSV-treated medulloblastoma tumors revealed expansion and activation of tumor-infiltrating dendritic cells (DCs), and direct oHSV infection of DCs within the brain. While the therapeutic effects of oHSVs have been primarily attributed to tumor cell infection, we hypothesize that direct infection of DCs also contributes to therapeutic efficacy by promoting DC maturation and immune activation. Although the oHSV infection in DCs was abortive, it led to increased expression of major histocompatibility complex (MHC) class I/II and co-stimulatory molecules. oHSV-infected DCs activated naïve CD4^+^ and CD8^+^ T cells, inducing expression of CD69 and CD25. These primed T cells exhibited enhanced cytotoxicity against CT-2A glioma cells. Adoptive transfer of oHSV-infected DCs via subcutaneous injection near inguinal lymph nodes delayed tumor growth in a syngeneic CT-2A glioma model, independent of tumor viral replication and lysis. Mechanistically, our in vitro studies demonstrate that oHSV can directly infect and functionally activate DCs, enabling them to prime effective anti-tumor T cell responses. This study highlights the anti-tumor potential of leveraging oHSV-infected DCs to augment viroimmunotherapy as a cancer therapeutic.

## 1. Introduction

Central nervous system (CNS) malignant tumors are associated with significant morbidity and mortality in both pediatric and adult patients [1]. Among them, malignant glioma (MG) is the most common primary CNS tumor with a median overall survival time of <2 years [2,3,4]. Medulloblastoma represents 20% of pediatric CNS tumors and carries an overall mortality rate of 30% [1,5,6]. Both tumors are characterized by a profoundly immunosuppressive tumor microenvironment (TME) [7,8]. Conventional treatment for malignant glioma consists of maximum safe total surgical resection and radio- and temozolomide chemotherapy, but these treatment-resistant tumors recur locally and ultimately lead to patient death [9], highlighting the need for novel therapeutic approaches.

Oncolytic herpes simplex virus (oHSV) has emerged as a promising experimental therapy for CNS tumors [10,11,12]. oHSV mediates direct tumor cell lysis and induces immunogenic cell death, thereby promoting anti-tumor immune responses. Early-stage clinical trials have demonstrated its safety and therapeutic potential in patients with malignant CNS tumors [13,14,15]. We previously reported the anti-tumor efficacy of a chimeric second-generation oHSV in which both copies of the principal neurovirulence gene, γ134.5, have been deleted from HSV-1 and it expresses the human cytomegalovirus (HCMV) IRS1 gene [16]. This modification enhances viral replication within tumors and improves therapeutic efficacy in murine tumor models. Notably, even in virus-resistant tumor models such as the DBT murine glioma cell line, where viral replication is limited, oHSV can still modulate immune responses and contribute to tumor control [17]. Thus, the oHSV-induced immune response is considered essential for durable anti-tumor activity [18,19].

Dendritic cells (DCs) are specialized antigen-presenting cells (APCs) and key elements of the TME. DCs can contribute to the activation of antitumor T-cell responses by infiltrating tumors, processing tumor-derived antigens, and presenting them to naïve T cells [20,21]. Within the TME, various tumor- and stromal-derived factors disrupt DC function, affecting their differentiation, maturation, antigen presentation, and longevity [22,23]. To this end, enhancing the recruitment of functional DCs and promoting DC-mediated anti-tumor immune responses has become an area of growing interest in the development and optimization of immunotherapies [24,25]. Talimogene laherparepvec (T-VEC) was the first oHSV approved by the US FDA for patients with metastatic melanoma [26]. This oHSV expresses GM-CSF which can recruit DCs and promote their maturation and antigen-specific T cell priming [27]. Based on our gene expression studies in subjects with recurrent GBMs who were treated with a fractional oHSV dose, there was a significant increase in the overall DC score and abundance of DCs relative to the total tumor-infiltrated lymphocyte (TIL) ratio in subjects with prolonged survival [18].

To determine if direct DC infection by oHSV could mediate a component of oHSV’s tumor therapeutic activity, we reanalyzed RNAseq-gene expression results from a published Phase Ib trial and our single cell RNA seq results from a murine syngeneic medulloblastoma model focusing on DC markers of activation and maturation. These results showed oHSV gene expression in infected DCs was associated with DC maturation and oHSV therapeutic activity. Based on these findings, we hypothesized that direct DC infection by oHSV promotes DC activation, contributing to the malignant glioma therapeutic activity. Here, we show that oHSVs can directly infect DCs, transiently express viral proteins (<24 h), and modulate DC maturation. Using infection and co-culture studies, we demonstrate that oHSV-infected DCs activate T cells and elicit anti-tumor effects. Next, to test whether direct oHSV-infected DCs could suppress tumor growth independent of virus mediated tumor cell lysis, immune competent mice were administered oHSV-infected DCs and then challenged with syngeneic glioma (CT-2A) tumors. These studies showed that mice pretreated with oHSV-infected DCs produced a local anti-tumor immune response capable of suppressing tumor growth. Collectively, our findings suggest that oHSV-infected DCs enhance anti-tumor immunity through T cell priming independent of cancer cell associated oncolytic virus replication and cell lysis.

## 2. Materials and Methods

### 2.1. Cells and Viruses

JAWSII cells, an immortalized DC line derived from C57BL/6 murine bone marrow, were obtained from the American Type Culture Collection (ATCC, CRL-2612). JAWSII cells were cultured in RPMI-1640 medium supplemented with 10% fetal bovine serum (FBS) and 5 ng/mL murine granulocyte-macrophage colony-stimulating factor (GM-CSF). Primary bone marrow-derived DCs (BMDCs) were generated from bone marrow isolated from the femurs and tibias of C57BL/6 mice. Following red blood cell lysis using ammonium-chloride-potassium (ACK) lysis buffer, the cells were cultured in RPMI-1640 medium containing 10% FBS, 20 ng/mL murine GM-CSF (BioLegend, San Diego, CA, USA), and 5 ng/mL murine interleukin-4 (IL-4, BioLegend, San Diego, CA, USA). Immature BMDCs were collected as non-adherent, floating cells on day 3 of culture. CT-2A cells were kindly provided by Dr. Thomas Seyfried (Boston College, MA, USA). Both parental CT-2A and luciferase-expressing CT-2A (CT-2A-luc) cells were cultured in Dulbecco’s Modified Eagle Medium (DMEM) supplemented with 2 mM L-glutamine, 4.5 mg/mL glucose, and 10% FBS. Vero cells (ATCC, Manassas, VA, USA) were used for virus production and titration and were cultured in DMEM supplemented with 5% bovine growth serum. All cell lines were maintained at 37 °C in a humidified incubator with 5% CO_2_. Viruses used in this study were described previously and all express eGFP including M2001 (wild-type [WT]-HSV + eGFP), C101 (first-generation oHSV; Δγ_1_34.5+eGFP), and C154 (second-generation oHSV; chimeric Δγ_1_34.5, HCMV IRS1 + eGFP) [16,28].

### 2.2. Flow Cytometry

Cells were seeded in 24-well plates and infected with WT-HSV, first-generation oHSV (C101), or second-generation oHSV (C154) at equivalent multiplicities of infection (MOI) and samples harvested at 8, 16, 24, or 48 h post infection (hpi). At the time of harvest, cells were washed with phosphate-buffered saline (PBS) and stained using Horizon™ Fixable Viability Stain 780 (BD Biosciences, Franklin Lakes, NJ, USA) for 15 min at 37 °C to identify dead cells. Samples were then incubated with TruStain FcX™ (anti-mouse CD16/32) to block Fc receptors for 5 min, followed by surface marker staining for 40 min at room temperature. After staining, cells were washed and resuspended in FACS buffer (PBS containing 5% FBS and 0.1% NaN_3_). Flow cytometry data were acquired using an Attune NxT flow cytometer (Thermo Fisher Scientific, Waltham, MA USA) and analyzed with FlowJo software (version 10.8.1; BD Biosciences, Franklin Lakes, NJ, USA).

The following antibodies (all from BioLegend, San Diego, CA, USA) were used for surface staining: Brilliant Violet 421 anti-mouse F4/80 (BM8), Brilliant Violet 510 anti-mouse I-A/I-E (M5/114.152), Brilliant Violet 605 anti-mouse Cluster of differentiation 45 (CD45) (30-F11), Brilliant Violet 650 anti-mouse CD80 (16-10A1), Brilliant Violet 785 anti-mouse CD11c (N418), PerCP/Cyanine5.5 anti-mouse CD11b (M1/70), PE/Dazzle594 anti-mouse CD86 (GL-1), PE/Cy7 anti-mouse CD40 (3/2.3), and Alexa Fluor 700 anti-mouse H-2Kb (AF6-88.5).

### 2.3. Viral Recovery and IncuCyte^®^ Viral Spread Assay

JAWSII cells and primary BMDCs were infected at MOI of 1, as described above. Cells were harvested at 2 hpi and washed twice with PBS to remove unbound virus then cultured in appropriate medium for an additional 24 or 48 h. Viral recovery was assessed by performing a limiting dilution plaque assay on Vero cells, as previously described [28]. For the viral spread assay, CT-2A cells (1 × 10^5^ cells/well) were seeded into 24-well plates, and infected with the virus, PBS-washed JAWSII cells (1 × 10^5^ cells/well) were subsequently added. Plates were monitored for 72 h using the IncuCyte^®^ SX5 live-cell imaging system (Sartorius, Göttingen, Germany). Images were acquired using a 10× objective and analyzed for phase contrast and GFP fluorescence using IncuCyte^®^ Cell-by-Cell Analysis Software Module.

### 2.4. Co-Culture Experiment and Tumor Killing Assay

Spleens were collected from non-tumor-bearing C57BL/6 mice, and CD3^+^ T cells were isolated using the MojoSort™ Mouse CD3 T Cell Isolation Kit (BioLegend, San Diego, CA, USA). As described above, JAWSII cells were treated with PBS, oHSV (C154), or WT-HSV (MOI = 1), washed twice with PBS at 2 hpi, and then co-cultured with splenic CD3^+^ T cells in RPMI-1640 medium supplemented with 10% FBS and 50 μM β-mercaptoethanol. Co-cultures were incubated for 24 and 48 h prior to analysis. As a stimulated control, T cells were activated with 2 μg/mL anti-mouse CD3 (BioLegend, San Diego, CA, USA), 1 μg/mL anti-mouse CD28 (BioLegend, San Diego, CA, USA), and 10 ng/mL recombinant mouse IL-2 in complete medium. After co-culture, T cell activation was assessed by staining for surface markers and flow cytometry, as described in Section 2.2. CD3^+^ T cells were subsequently isolated from the culture suspension and used for the tumor cell killing assay.

CT-2A-luc (2 × 10^4^ cells/well) were seeded in 96-well plates and allowed to adhere overnight. The following day, purified CD3^+^ T cells were then added at an effector to target (E:T) ratio of 5:1 (1 × 10^5^ cells/well). The plates were placed in an IncuCyte^®^ SX5 live-cell imaging system (Sartorius, Göttingen, Germany) and monitored for 48 h. After incubation, cells were lysed using the One-Glo Luciferase Assay System (Promega, Madison, WI, USA) according to the manufacturer’s instructions. Lysates were transferred to a white 96-well plate, and bioluminescence was measured using a Synergy 2 plate reader (BioTek, Winooski, VT, USA).

### 2.5. Syngeneic Murine Model

All animal studies were approved by the Institutional Animal Care and Use Committee (IACUC) of Nationwide Children’s Hospital (protocol number AR16-00057) and the NIH Guide for the Care and Use of Laboratory Animals. For DC tracking studies, DCs were labeled with Vybrant™ DiD Cell-Labeling Dye (Thermo Fisher Scientific, Waltham, MA USA) following the manufacturer’s protocol. Labeled DCs were administered via intraperitoneal (IP), intratumoral (IT), or subcutaneous (SC; inguinal region) injection into mice bearing flank tumors measuring 50–100 mm^3^. Each cohort consisted of five mice, including one mouse that received unstained DCs as a negative control. Fluorescent imaging was performed on day 1 post-injection and on day 4 after euthanasia using the Xenogen IVIS Spectrum imaging system (Caliper Life Sciences, Hopkinton, MA, USA).

For CT-2A flank tumor studies, 5- to 6-week-old C57BL/6 mice (Envigo) received subcutaneous injections of vehicle or 1 × 10^4^ PBS- or oHSV-treated DCs (MOI = 10, 16 hpi) in 50 µL of serum-free DMEM into the left inguinal region. DC injections were administered twice, one week apart. One week following the second DC injection, CT-2A cells (1 × 10^6^ cells in 50 µL serum-free DMEM) were implanted subcutaneously into each flank. Tumor growth was monitored weekly using calipers, and tumor volume was calculated. Mice were euthanized once tumors reached IACUC-defined endpoint criteria: 3000 mm^3^ total tumor volume per mouse or 2000 mm^3^ per individual tumor. Flank tumor growth experiments were independently repeated to confirm biological reproducibility. Sample sizes were as follows: non-DC control (*n* = 8 mice), PBS-DC (*n* = 12 mice), and oHSV-DC (*n* = 12 mice).

### 2.6. Statistical Analysis

All data were analyzed using GraphPad Prism software (version 10.3.1). Results are presented as mean ± standard error of the mean (SEM) for tumor growth curves. Comparisons between two or more groups were performed using one-way or two-way ANOVA, followed by appropriate multiple comparison tests as indicated in figure legends. Statistical significance is indicated as follows: * *p* < 0.05, ** *p* < 0.01, *** *p* < 0.001, **** *p* < 0.0001.

## 3. Results

### 3.1. Secondary Analysis of Pre-Clinical Results Suggests That oHSV Infection Promotes DC Maturation

**Figure 1:**
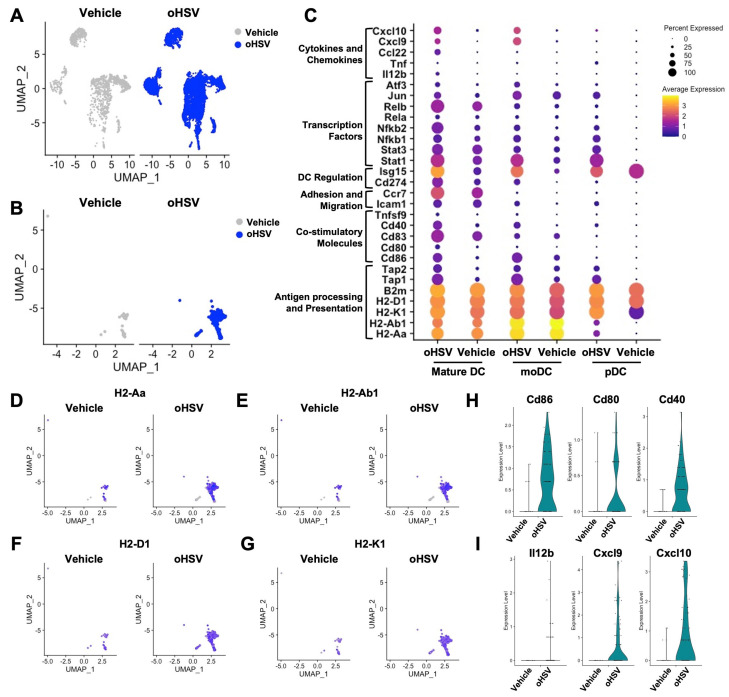
Single-cell RNA-seq (scRNA-seq) analysis of tumor-infiltrating dendritic cells (DCs) reveals DC maturation following oncolytic herpes simplex virus (oHSV) treatment. UMAP projection of (**A**) tumor-infiltrating lymphocytes (TILs) and (**B**) DC subsets from vehicle-treated (gray) and oHSV-treated (blue) mice analyzed by scRNA-seq (*n* = 8). (**C**) Differentially expressed genes associated with DC activation in mature DCs, monocyte-derived DCs (moDCs), and plasmacytoid DCs (pDCs) from vehicle- and oHSV-treated mice. UMAP plots comparing expression of major histocompatibility complex (MHC) class II and I genes: (**D**) *H2-Aa*, (**E**) *H2-Ab1*, (**F**) *H2-D1*, and (**G**) *H2-K1* split by treatment group. Violin plots showing expression levels of (**H**) co-stimulatory molecules and (**I**) key cytokines/chemokines.

Our previous studies involving Phase IB clinical trial samples from oHSV-treated subjects demonstrated that oHSV treatment increased the number of tumor-infiltrating DCs and antigen processing and presentation [18]. To evaluate whether oHSV treatment has a similar impact in our murine pre-clinical models, we performed a secondary analysis on our single cell RNA sequencing (scRNA-seq) dataset from another C57BL/6 syngeneic CNS malignancy (medulloblastoma) model [29]. The prior studies demonstrated that oHSV treatment enhances general leukocyte migration to the brain (Figure 1A) and focused on T cell and monocyte/macrophage changes post-oHSV treatment. In this reanalysis, we concentrated specifically on DC subpopulations, their activation status, and maturation, and also identified tumor-infiltrating DC expansion post-oHSV-treatment in mice compared to vehicle-treated controls (Figure 1B). We next examined the effects of oHSV treatment on DC activation by assessing genes involved in antigen processing and presentation (*H2-Aa*, *H2-Ab1*, *H2-K1*, *H2-D1*, *B2m*, *Tap1*, *Tap2*), co-stimulatory signaling (*Cd86*, *Cd80*, *Cd83*, *Cd40*, *Tnfsf9*), adhesion and migration (*Icam1*, *Ccr7*), immune regulation (*Cd274*, *Isg15*), transcriptional regulation (*Stat1*, *Stat3*, *Nfkb1*, *Nfkb2*, *Rela*, *Relb*, *Jun*, *Atf3*), and cytokine/chemokine production (*Il12b*, *Tnf*, *Ccl22*, *Cxcl9*, *Cxcl10*) (Figure 1C). Our results show that oHSV treatment increases the expression of key immune markers in plasmacytoid DCs, monocyte-derived DCs, and most notably, mature DCs, compared to those in vehicle-treated mice. DCs from oHSV-treated tumors exhibited significantly higher expression of major histocompatibility complex (MHC) class II molecules (*H2-Aa* and *H2-Ab1*) and class I molecules (*H2-D1* and *H2-K1*) (Figure 1D–G), co-stimulatory markers (*Cd86*, *Cd80*, and *Cd40*), and cytokines/chemokines such as *Il12b*, *Cxcl9*, and *Cxcl10* (Figure 1H,I), relative to DCs from vehicle-treated mice. These findings were consistent with our Phase IB clinical study and suggest that oHSV has the potential to promote the maturation of otherwise immature DCs within the murine malignant brain tumor microenvironment. Notably, we previously published detection of oHSV gene expression within DCs at day 2 post-treatment, suggestive of direct viral infection of the DC population [29]. While oHSV, as a conditionally replication-competent virus, selectively replicates and enhances gene expression in tumor cells, this observation raises the possibility that direct infection of DCs may contribute to the immunotherapeutic activity of oHSV within the TME.

### 3.2. oHSV Can Directly Infect DCs but oHSV Replication Is Restricted

**Figure 2:**
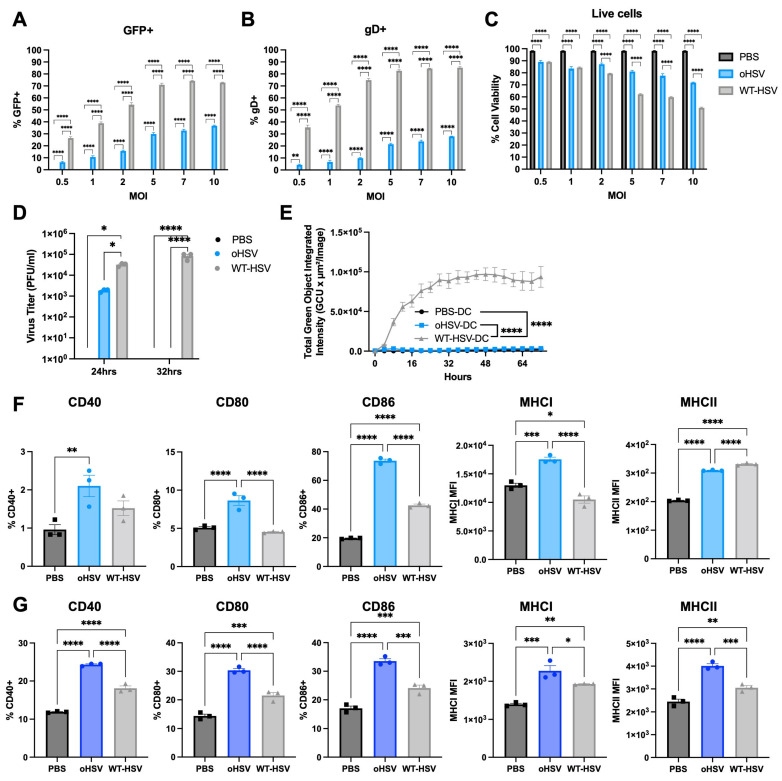
Direct infection of DCs by oHSV and assessment of DC maturation in vitro. (**A**) Green fluorescent protein (GFP) expression, (**B**) herpes simplex virus (HSV) glycoprotein D (gD) expression, and (**C**) live cell counts in DCs following treatment with PBS (black), second-generation oHSV (hereafter referred to as oHSV; blue), or wild-type-HSV (WT-HSV) (gray) at indicated multiplicities of infections (MOIs). (**D**) Viral recovery assay from DCs at 24 and 32 h post-infection with PBS, oHSV, or WT-HSV (MOI = 1). (**E**) Viral spread assay in CT-2A cells co-cultured with PBS-, oHSV-, or WT-HSV-treated DCs (MOI = 1), analyzed using the Incucyte live-cell imaging system. (**F**) Surface expression of co-stimulatory molecules and major histocompatibility complex (MHC) class I and II in PBS-, oHSV-, or WT-HSV-treated immortalized DCs (MOI = 1). (**G**) Surface expression of the same markers in primary bone marrow-derived DCs (BMDCs) treated under identical conditions. Results are shown as mean ± standard error of the mean (SEM) with each point representing a single replicate. Statistical significance was determined using two-way ANOVA test with Tukey’s multiple comparisons (**A**–**D**) and ordinary one-way ANOVA test with Holm–Šídák’s multiple comparisons test (**E**–**G**). * *p* < 0.05, ** *p* < 0.01, *** *p* < 0.001, **** *p* < 0.0001.

Based on the detection of oHSV gene expression and increased expression of maturation genes within the DC population in oHSV-treated mice, we hypothesized that oHSVs could directly infect DCs and modulate their maturation. To test this, we treated immortalized bone marrow-derived DCs with PBS, WT-HSV, first-generation oHSV (Δγ_1_34.5), or second-generation oHSV (Δγ134.5 HCMV IRS1 gene) at different MOIs (0.5, 1, 2, 5, 7, or 10 plaque-forming units [pfu]/cell). All HSV constructs used in this study encoded enhanced green fluorescent protein (EGFP) to facilitate detection of infection. We assessed both GFP and HSV glycoprotein D (gD) surface expression and cell viability at 16 hpi by flow cytometry (Figure 2A–C and Figure S1A,B). Second-generation oHSV-treated DCs exhibited approximately 10–15% higher proportion of GFP expression and 5% higher gD expression than first-generation oHSV-treated cells in the DCs (Figure S1A,B). Given its enhanced viral gene expression within DCs and relevance to ongoing clinical translation, we selected the second-generation oHSV (hereafter referred to as oHSV) for further analysis.

As shown in Figure 2A,B, both GFP and gD expression increased in a dose-dependent manner with rising MOI, indicating effective DC infection and sustained viral gene expression up to 16 hpi. At MOIs > 5, oHSV treatment modestly affected DC viability, though ~72% of cells remained viable. In contrast, WT-HSV infection caused significantly greater cytotoxicity, with only 50% of cells remaining viable at MOI 10 (Figure 2C). Based on comparable infection rates (15–20%) and differential cytotoxicity profiles, we selected MOI 1 for subsequent experiments.

Second-generation oHSV (oHSV) is a conditionally replication-competent oHSV, and previous studies have demonstrated its selective tumor-associated replication [30]. To verify that oHSV DC infection was non-productive, we performed viral recovery studies from infected DCs at 24 and 32 hpi. WT-HSV viral production increased over 32 hpi in the DCs (24 hpi: 3 × 10^4^ pfu/mL, 32 hpi: 8.3 × 10^4^ pfu/mL). In contrast, less virus was recovered at 24 hpi in the oHSV-infected DCs (1.8 × 10^3^ pfu/mL), and no virus was recovered by 32 hpi (Figure 2D). To ensure that the virus was not present below the threshold of detection, we co-cultured PBS- (PBS-DC), oHSV- (oHSV-DC) or WT-HSV-treated DC (WT-HSV-DC) with CT-2A murine malignant glioma cells and evaluated viral GFP expression using the IncuCyte live cell imaging system. While CT-2As co-cultured with WT-HSV-DCs demonstrated GFP production (75160) in the incubated CT-2As, oHSV-DCs co-cultured with CT-2As did not produce detectable GFP and did not significantly differ from PBS-DCs (PBS-DC 788.4 vs. oHSV-DC 2100, *p* = 0.8145) (Figure 2E and Figure S1C). These results indicate that DCs are susceptible to oHSV infection and transient transgene expression for a brief period post-infection. However, this infection is abortive and showed no significant detectable virus production by 32 hpi in contrast to WT-HSV, which produces increased viral progeny through ongoing replication.

### 3.3. oHSV Infection Alters DC Maturation

As DC activation is critical for initiating immune responses, and our earlier clinical and pre-clinical studies suggest that oHSV alters DC maturation, we next examined the impact of oHSV infection and transient viral gene expression on DC maturation. To assess DC maturation in vitro, we measured the surface expression of co-stimulatory (CD40, CD80, and CD86) and MHC class I and II molecules on immortalized bone marrow-derived DCs using flow cytometry.

Consistent with our scRNA-seq dataset, oHSV-DCs showed increased surface expression of all co-stimulatory markers (CD40, CD80, and CD86) as well as MHC class I molecules at 24 and 48 hpi compared to PBS-DCs (Figure 2F and Figure S1D). Although WT-HSV-DCs also upregulated these markers relative to PBS-DCs, their expression levels were similar to or lower than those observed in oHSV-DCs (Figure 2F). These results suggest that oHSV promotes DC activation and drives the phenotypic shift from an immature to a mature state following infection.

To determine whether the findings from immortalized DCs could be recapitulated in primary bone marrow-derived dendritic cells (BMDCs), we repeated the experiments using primary BMDCs and analyzed surface expression of co-stimulatory molecules and MHC class I and II surface expression after oHSV treatment. As shown in Figure S1F, because primary BMDCs are more resistant to oHSV infection than are immortalized DCs, we used a MOI of 2, which achieved GFP expression level (~10%) to that observed in the immortalized DC studies. Consistent with those findings, oHSV infection of primary BMDCs increased CD40, CD80, CD86, and MHC class I and II molecules surface expression compared to PBS- or WT-HSV-treated BMDCs at 48 hpi (Figure 2G).

### 3.4. oHSV-Infected DCs Can Prime T Cells That Can Mediate a Functional Anti-Tumor Response

**Figure 3:**
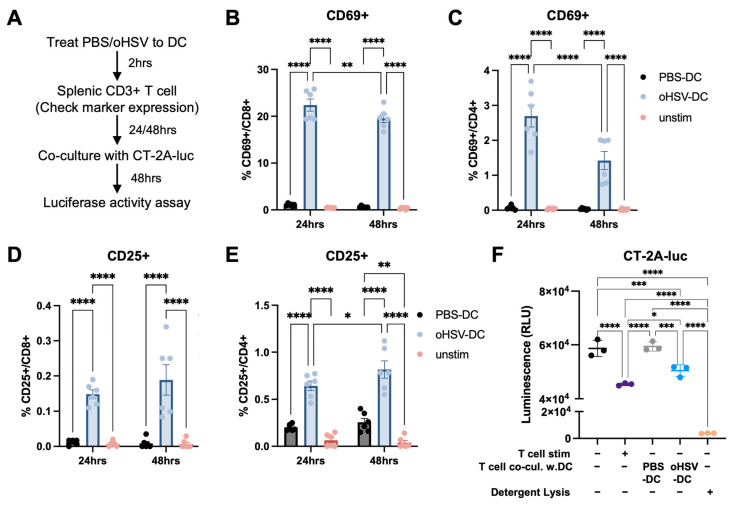
T cell activation induced by oHSV-treated DCs. (**A**) Schematic of the co-culture experiment using splenic T cells. Expression of activation markers CD69 on (**B**) CD8^+^ and (**C**) CD4^+^ T cells after co-culture with PBS- (black) or oHSV-treated DCs (blue) or unstimulated (pink). Expression of CD25 on (**D**) CD8^+^ and (**E**) CD4^+^ T cells under the same conditions. Results are shown as mean ± SEM, combined from two independent experiments (*n* = 6), with each point representing a single replicate. (**F**) Luciferase activity assay using CT-2A expressing luciferase (CT-2A-luc) cells (2 × 10^4^ cells/well) cultured with T cells (1 × 10^5^ cells/well) for 48 h under the following conditions: unstimulated T cells; T cells stimulated with anti-CD3 and CD28 (T cell stim); or T cells pre-co-cultured for 16 h with either PBS-treated DCs (PBS-DCs) or oHSV-treated DCs (oHSV-DCs). As a reference for the lowest luminescence level, CT-2A-luc cells were detergent-lysed (Detergent lysis). Data are shown as mean ± SEM with each point representing a single replicate. Two-way ANOVA with Tukey’s multiple comparisons test (**B**–**E**) and ordinary one-way ANOVA test with Holm-Šídák’s multiple comparisons test (**F**) were used for statistical analysis. * *p* < 0.05, ** *p* < 0.01, *** *p* < 0.001, **** *p* < 0.0001.

DCs play a central role in initiating and sustaining antiviral and antitumor immune responses and are considered key mediators bridging innate and adaptive immunity [20]. Previous studies have shown that T cells can be activated in an antigen-independent manner through cytokine signaling during viral infection, allowing them to contribute to immune responses by producing effector cytokines and mediating cytotoxicity [31]. To evaluate whether oHSV-DCs are capable of priming T cells, we co-cultured them with naïve splenic CD3^+^ T cells for 24 or 48 h and assessed T cell activation marker expression (Figure 3A). CD3^+^ T cells co-cultured with oHSV-DCs exhibited increased CD69 expression, and this early activation marker was present on both CD8^+^ and CD4^+^ T cells at both 24 and 48 h compared to the PBS-DC controls (Figure 3B,C). CD25, a late activation marker, was also significantly upregulated on CD8^+^ and CD4^+^ T cells after oHSV-DC co-culture (Figure 3D,E).

Recent studies have shown that antiviral immune responses can enhance antitumor immunity and may help identify therapeutic targets to improve responses to immunotherapy [32]. To assess the impact of oHSV-DCs on T cell-mediated tumor killing, we co-cultured luciferase-expressing CT-2A cells (CT-2A-luc) with CD3^+^ T cells that had been previously co-cultured with either PBS-DCs or oHSV-DCs. Using a luciferase assay and the IncuCyte^®^ live-cell imaging system, T cells co-cultured with PBS-DCs showed no significant difference in cytotoxicity relative to unstimulated T cells alone. In contrast, T cells co-culture with oHSV-DCs led to significantly greater cytotoxicity against CT-2A-luc cells compared to both T cells co-cultured with PBS-DCs (*p* = 0.0006) and unstimulated T cell controls (*p* = 0.0008) (Figure 3F and Figure S2). These findings indicate that oHSV-activated DCs play a critical role in priming CD4^+^ and CD8^+^ T cells and enhancing their tumor-killing activity against CT-2A tumors.

### 3.5. oHSV-Infected DC Mediates Therapeutic Effect Against Malignant Brain Tumor

Having established that oHSV-DCs can prime T cells and enhance T cell–mediated tumor killing in vitro, we next sought to determine whether oHSV-DCs could mediate an anti-tumor response in vivo, independent of viral replication and tumor antigen release. To test this, we adoptively transferred oHSV-DCs into mice and evaluated their ability to suppress CT-2A tumor establishment.

Since mature DCs migrate to draining lymph nodes (dLNs) to interact with and prime T cells, we hypothesized that enhancing DC migration to dLNs would improve antitumor efficacy. We employed a flank tumor model to enable a direct examination of the treated tumor flanks and dLNs. To identify the optimal route of DC administration, we compared DC migration following intraperitoneal (IP), intratumoral (IT), and inguinal subcutaneous (SC) injection (Figure S3A). Using DiD fluorescent-labeled DCs, we observed that SC injection into the right inguinal region resulted in selective accumulation of DCs in the corresponding dLN. In contrast, IP and IT injections led to broader systemic distribution, with prominent DiD signals detected in the spleen and liver at day 4 post-injection (Figure S3B). These results support inguinal SC injection as the preferred route for delivering oHSV-DCs to promote localized immune activation.

**Figure 4:**
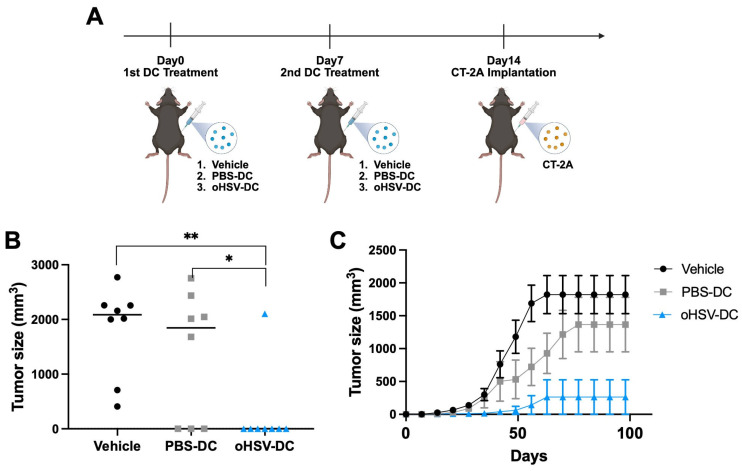
Therapeutic efficacy of oHSV-infected DCs in a malignant brain tumor model. (**A**) Schematic of the in vivo study design. (**B**) CT-2A tumor volumes at endpoint for individual mice in each treatment group: vehicle (black), PBS-DC (gray), and oHSV-DC (blue) each point represents one mouse (*n* = 8 per cohort). (**C**) CT-2A tumor growth curves in mice receiving vehicle, PBS-DC, or oHSV-DC. Data are presented as median (**B**) or mean ± SEM (**C**). Statistical analysis was performed using ordinary one-way ANOVA followed by Holm–Šídák’s multiple comparisons test. * *p* < 0.05, ** *p* < 0.01.

As shown in Figure 4A, we employed a prime–boost immunization strategy by administering oHSV-DCs twice, one week apart, prior to tumor implantation. To evaluate the therapeutic effect of adoptively transferred oHSV-DCs on local tumor growth, we subcutaneously injected either 1 × 10^4^ PBS-DCs or oHSV-DCs into one side of the inguinal region on Day0 and again on Day7. One week after the second DC administration (Day14), mice were implanted with 1 × 10^6^ CT-2A cells on the same side as the DC treatment (Figure 4A). Both the tumor sizes at the endpoint and the tumor growth curves demonstrate that mice treated with oHSV-DCs exhibited significantly delayed tumor growth compared to vehicle treated or PBS-DCs treated mice (Figure 4B,C). Taken together, these findings suggest that oHSV-DCs can suppress tumor growth through localized immune activation, supporting their potential as an effective adoptive cell therapy.

## 4. Discussion

In this study, we investigated the effects of direct oHSV infection of DCs. Our previous analysis of patient samples from a Phase IB clinical trial demonstrated an increased number of intratumoral DCs following oHSV treatment compared to pre-treatment levels [18]. Although earlier studies have reported changes in TILs, including DC populations, in response to oHSV, these efforts have primarily focused on immune activation driven by oHSV infection of tumor cells [18,29,33,34]. Notably, our scRNA-seq analysis of a murine medulloblastoma model revealed oHSV gene expression not only in tumor cells but also within DCs [29], suggesting direct viral infection of the DC population. Based on these findings, we hypothesized that enhancing DC function through oHSV infection may augment the therapeutic efficacy of oHSV-based treatments.

Both direct viral lysis and indirect immune-mediated tumor cell activity are considered integral to the virotherapy’s efficacy. Oncolytic viruses are engineered to selectively infect, replicate in, and lyse tumor cells [13,35]. This direct lysis not only reduces tumor burden but also is thought to release tumor associated antigens, which can be processed by APCs to facilitate antigen-specific immune responses [14,36]. In this study, we sought to evaluate whether direct viral infection of APC could also contribute to the immune-mediated antitumor activity independent of direct oncolytic activity. Here we show that oHSV infection of DCs leads to an abortive infection with transient viral gene expression that alters DC maturation and leads to immune-mediated tumor therapeutic effects. Our findings suggest that such an abortive infection could modulate DC functional activity within the tumor microenvironment. Further studies are needed to identify whether abortive oHSV infection specifically alters DC antigen presentation, potentially influencing adaptive memory immune responses and contributing to secondary changes in immune activity.

We treated both immortalized and primary BMDCs with oHSV and observed increased expression of MHC class I and II molecules, as well as co-stimulatory markers, in oHSV-DCs compared to PBS- or WT-HSV-DCs (Figure 2). Notably, CD86 and MHC class II expression were also elevated in WT-HSV–treated immortalized DCs, and expression of both co-stimulatory molecules and MHC class I/II was similarly upregulated in WT-HSV-treated primary BMDCs. Although WT-HSV has been reported to impair DC maturation and migration by degrading key molecules involved in these processes, thereby reducing T cell activation and proliferation [37,38], other studies have demonstrated that WT-HSV can also promote DC maturation and enhance antiviral cytokine production [39,40,41]. In our experiments, we used a MOI of 1 pfu/cell for both in vitro and in vivo studies, which preserved comparable cell viability between oHSV- and WT-HSV-DCs. Under these conditions, WT-HSV replication was insufficient to degrade maturation markers or induce DC lysis.

T cell activation can occur in both antigen-dependent and antigen-independent manners under viral treatment conditions [31,32]. In our study, co-culture with oHSV-DCs resulted in upregulation of T cell activation markers, and these activated T cells demonstrated enhanced CT-2A tumor cell killing (Figure 3). Given that oHSV does not replicate within DCs and that the CT-2A cells were never exposed to oHSV, it is unlikely that the observed T cell cytotoxicity occurred through antigen presentation pathways. Instead, these findings are suggestive of antigen-independent T cell cytotoxicity (e.g., cytokine production, Fas/L) likely driven by oHSV-DC derived cytokines. However, antigen spreading or T cell cross-reactivity may also contribute to the observed effects [42]. Further investigation is required to define the specific mechanisms by which oHSV-DCs activate T cells and drive downstream cytotoxicity.

DCs have been widely utilized as cellular therapeutics, such as in DC vaccination strategies using peptide or tumor lysate antigens across various cancer research fields [43,44,45]. In this study, we adoptively transferred oHSV-DCs into mice to investigate whether they contribute significantly to antitumor immunity in a syngeneic immunocompetent tumor model. We employed a flank tumor model allowing tracking of DC migration and assessment of local DC administration on tumor growth [46,47]. Using DiD-labeled DC migration experiments (Figure S3), we observed that DCs trafficked to the dLNs on the treated side, but not to LNs on the contralateral side, at day 4 post-injection. This suggests that DC migration may be restricted to localized bilateral lymphatic drainage at least at early time points. Notably, oHSV-DCs significantly reduced tumor growth at the injection site compared to PBS-DCs (Figure 4). These findings suggest that oHSV-DCs are sufficient to initiate localized immune responses but may not be capable of eliciting a systemic antitumor effect. However, future studies are needed to verify anti-tumor immune mechanism, especially adaptive immune system in vivo.

Murine CNS tumor models can recapitulate some aspects of human disease and provide important pre-clinical insights; however, they never fully replicate the complexity of human tumors [48,49,50]. For example, murine xenograft glioma models while helpful for evaluating human tumor associated direct oncolytic activity, they require immunocompromised animals, which limits the ability to assess immune-mediated responses of this therapy [51,52]. Likewise, while syngeneic murine models enable the evaluation of immune-mediated virotherapeutic effects, limited viral replication in these models remains a challenge and may not accurately reflect the improved virus replication in the human tumor. Here, using a syngeneic glioma model, these studies were designed to evaluate two questions: (1) whether DC infection is productive or abortive, and (2) whether this infection can alter DC maturation and subsequent immune-mediated antitumor therapeutic effects. To evaluate this functional immune activity, we used a tumor cell challenge assay rather than the treating established tumors. Therefore, this study does not address the complex effects of an established immunosuppressive tumor microenvironment, which could potentially override the impact of DC maturation. Further studies using orthotopic murine CNS tumor models are required to evaluate the immune-mediated antitumor response following DC treatment.

Taken together, our study demonstrates that oHSV can directly infect DCs, modulate their maturation, and subsequently induce antitumor immune responses through T cell activation. Furthermore, our findings demonstrate that oHSV-DCs are capable of reducing tumor growth in vitro, highlighting their potential as an effective adoptive cell therapy. Further studies are necessary to elucidate the precise mechanisms by which oHSV-DCs mediate antitumor activity. Furthermore, these studies do not address whether tumor associated immunosuppressive environment can override this OV associated DC maturation. These findings support continued investigation of adoptive oHSV-DC therapy as a promising therapeutic strategy for cancer.

## Data Availability

Data available in a publicly accessible repository. The original data presented in the study are openly available in NCBI’s Gene Expression Omnibus (GEO) under the Series accession number GSE200008.

## Supplementary Materials

The following supporting information can be downloaded at: https://www.mdpi.com/article/10.3390/v17081134/s1, Figure S1. Characterization of JAWSII and primary bone marrow-derived dendritic cells (BMDCs). Figure S2. T cell–mediated tumor cell killing activity. Figure S3. In vivo tracking of DC migration in a syngeneic immunocompetent tumor model.
