# Supplementary material for: Direct oHSV Infection Induces DC Maturation and a Tumor Therapeutic Response
Source: Viruses. 2025 Aug 19;17(8):1134. doi: 10.3390/v17081134 (PMC12390734; doi:10.3390/v17081134)
Supplement: Supplementary file 1 [file viruses-17-01134-s001.zip › viruses-3774533-supplementary.pptx]

## Slide 1
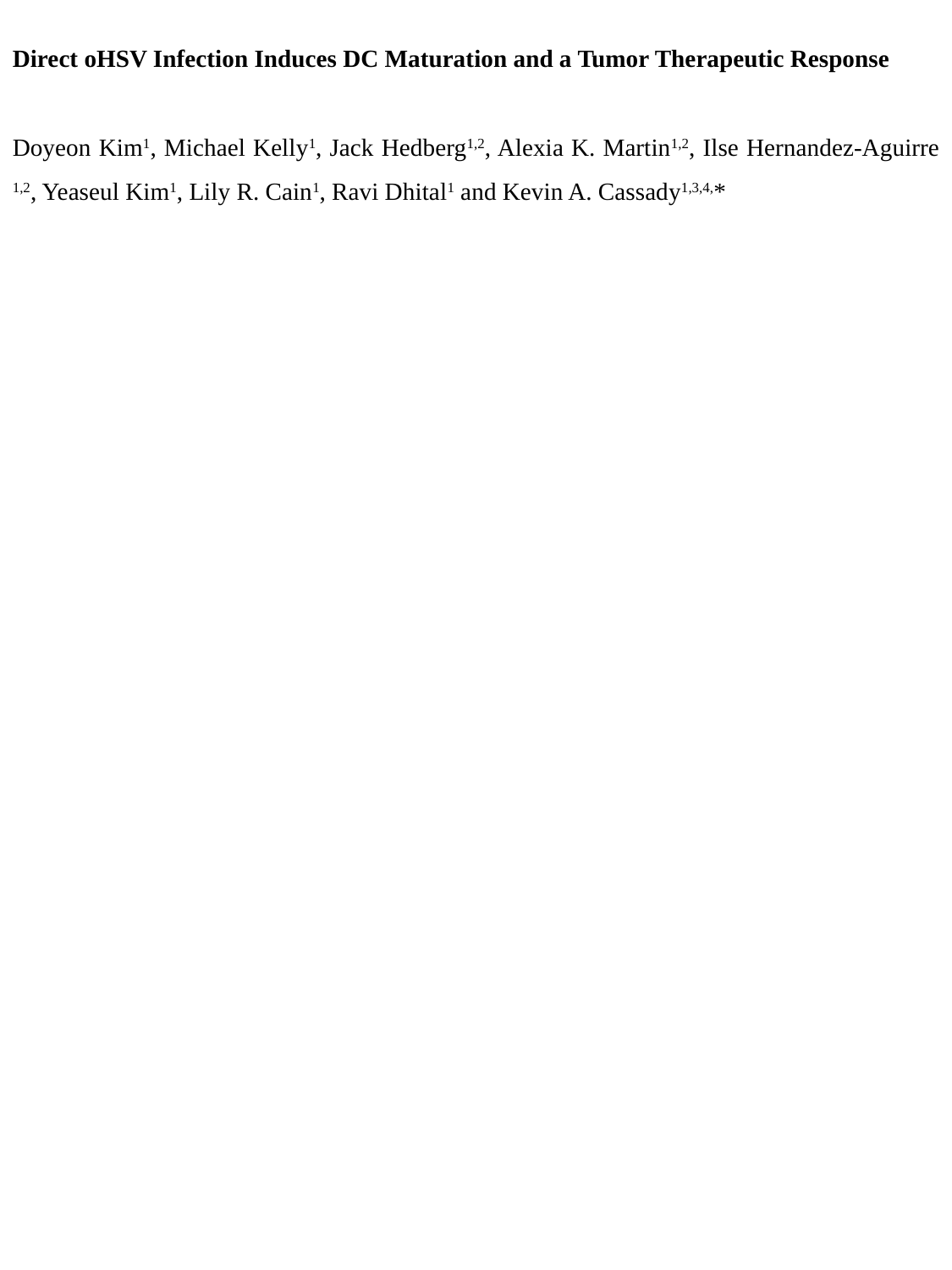

Direct oHSV Infection Induces DC Maturation and a Tumor Therapeutic Response
Doyeon Kim1, Michael Kelly1, Jack Hedberg1,2, Alexia K. Martin1,2, Ilse Hernandez-Aguirre 1,2, Yeaseul Kim1, Lily R. Cain1, Ravi Dhital1 and Kevin A. Cassady1,3,4,*

## Slide 2
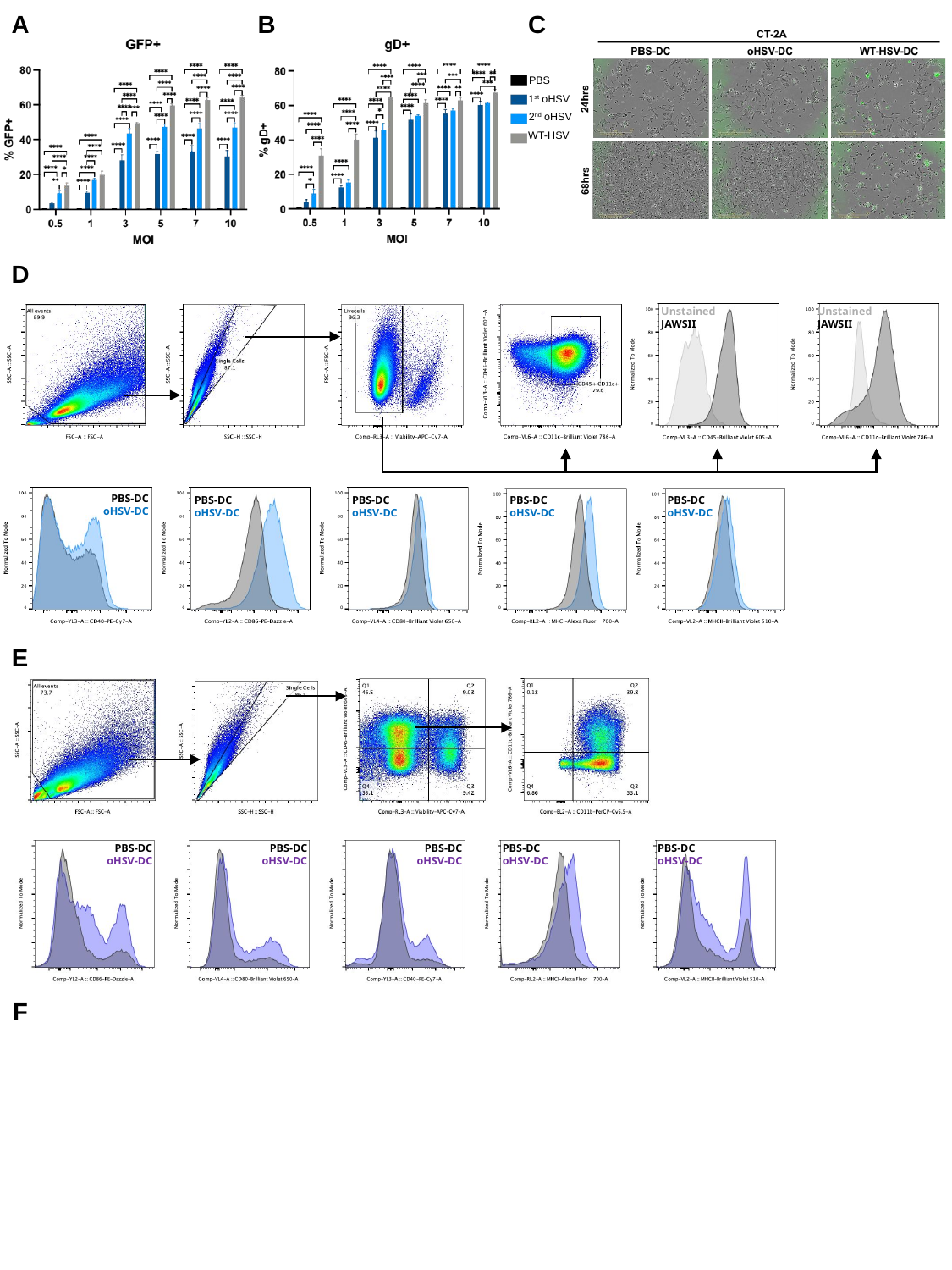

A
B
C
PBS
1st oHSV
2nd oHSV
WT-HSV
D
Unstained
JAWSII
Unstained
JAWSII
PBS-DC
oHSV-DC
PBS-DC
oHSV-DC
PBS-DC
oHSV-DC
PBS-DC
oHSV-DC
PBS-DC
oHSV-DC
E
PBS-DC
oHSV-DC
PBS-DC
oHSV-DC
PBS-DC
oHSV-DC
PBS-DC
oHSV-DC
PBS-DC
oHSV-DC
F

## Slide 3
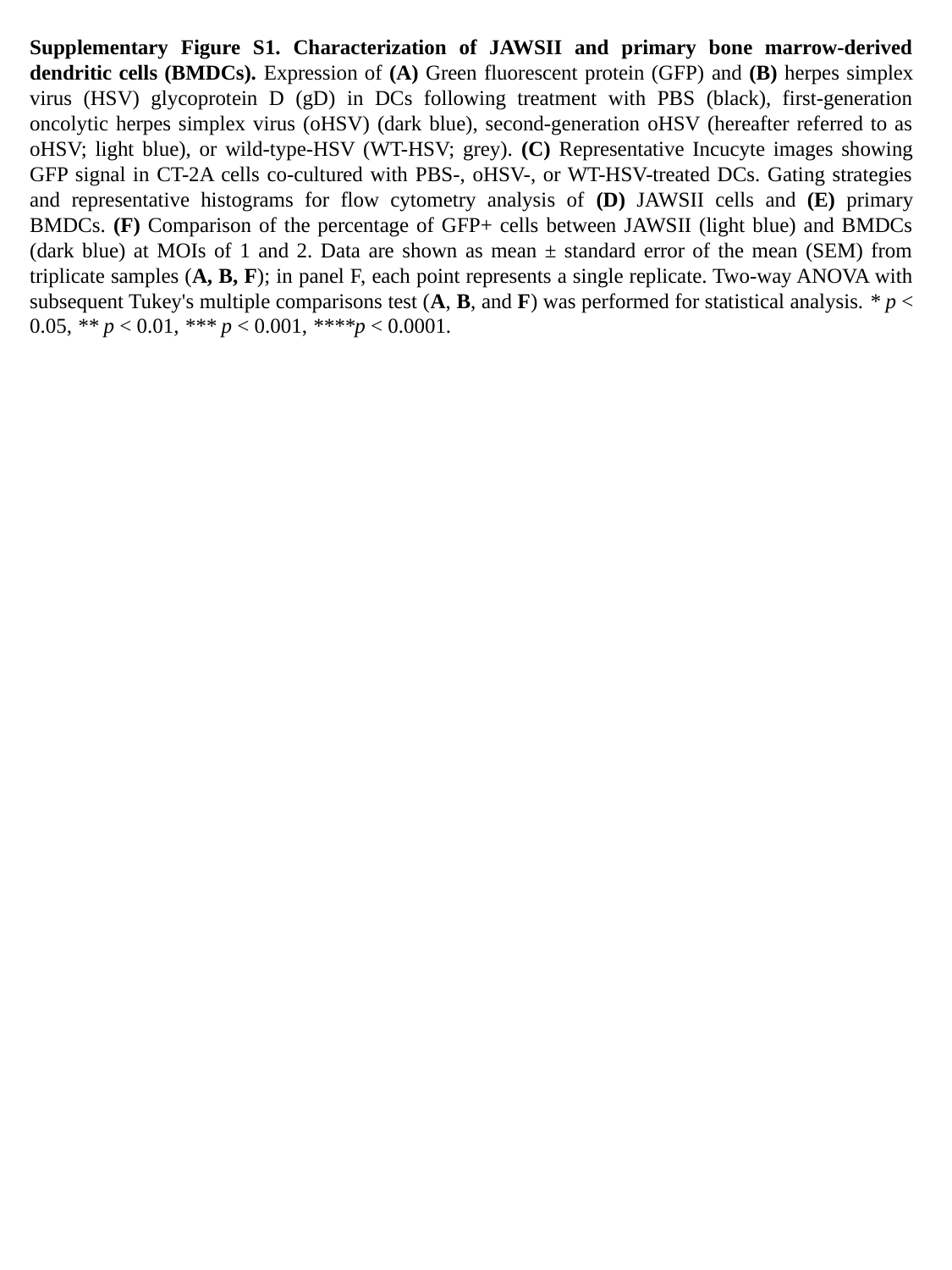

Supplementary Figure S1. Characterization of JAWSII and primary bone marrow-derived dendritic cells (BMDCs). Expression of (A) Green fluorescent protein (GFP) and (B) herpes simplex virus (HSV) glycoprotein D (gD) in DCs following treatment with PBS (black), first-generation oncolytic herpes simplex virus (oHSV) (dark blue), second-generation oHSV (hereafter referred to as oHSV; light blue), or wild-type-HSV (WT-HSV; grey). (C) Representative Incucyte images showing GFP signal in CT-2A cells co-cultured with PBS-, oHSV-, or WT-HSV-treated DCs. Gating strategies and representative histograms for flow cytometry analysis of (D) JAWSII cells and (E) primary BMDCs. (F) Comparison of the percentage of GFP+ cells between JAWSII (light blue) and BMDCs (dark blue) at MOIs of 1 and 2. Data are shown as mean ± standard error of the mean (SEM) from triplicate samples (A, B, F); in panel F, each point represents a single replicate. Two-way ANOVA with subsequent Tukey's multiple comparisons test (A, B, and F) was performed for statistical analysis. * p < 0.05, ** p < 0.01, *** p < 0.001, ****p < 0.0001.

## Slide 4
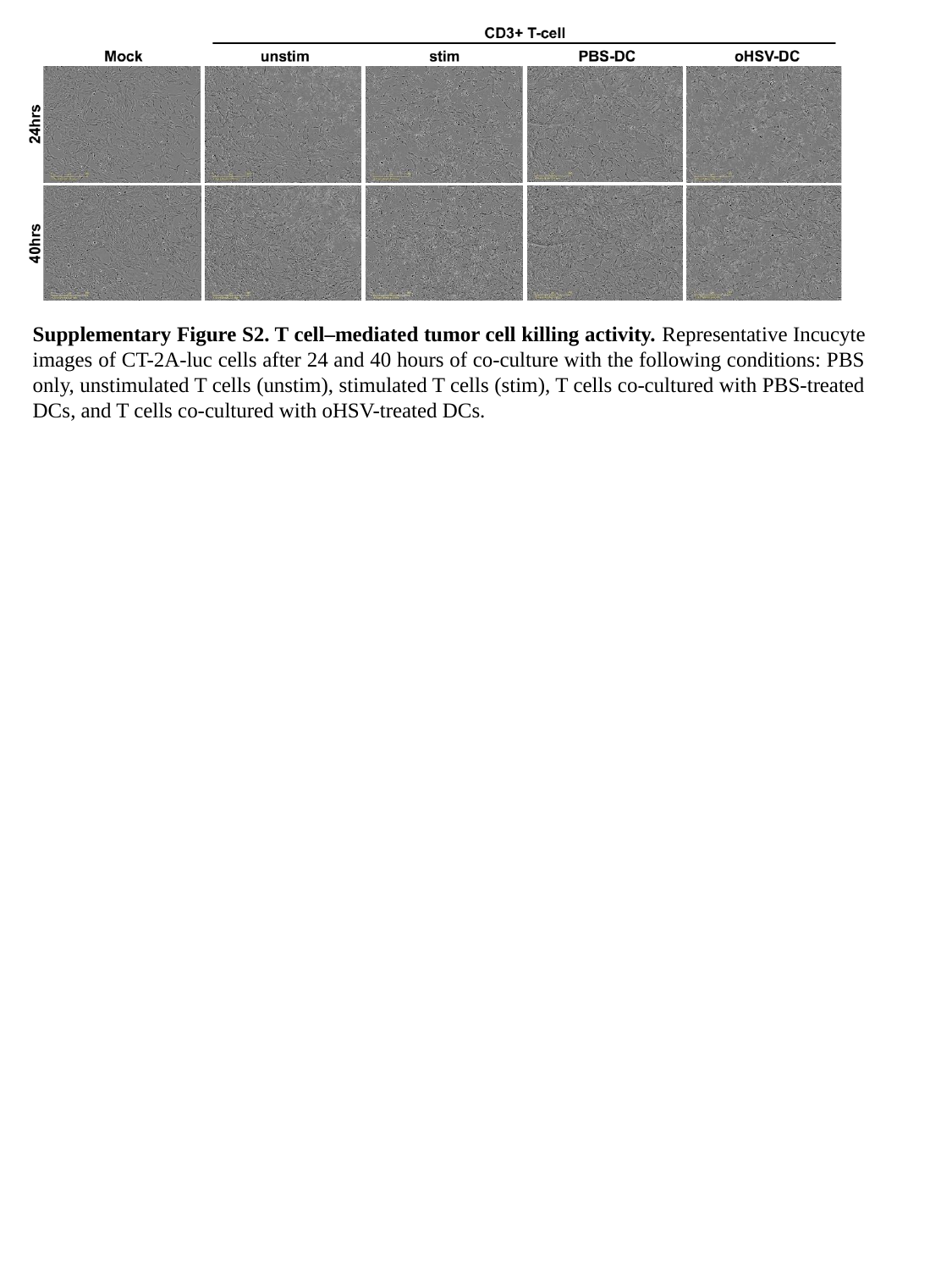

Supplementary Figure S2. T cell–mediated tumor cell killing activity. Representative Incucyte images of CT-2A-luc cells after 24 and 40 hours of co-culture with the following conditions: PBS only, unstimulated T cells (unstim), stimulated T cells (stim), T cells co-cultured with PBS-treated DCs, and T cells co-cultured with oHSV-treated DCs.

## Slide 5
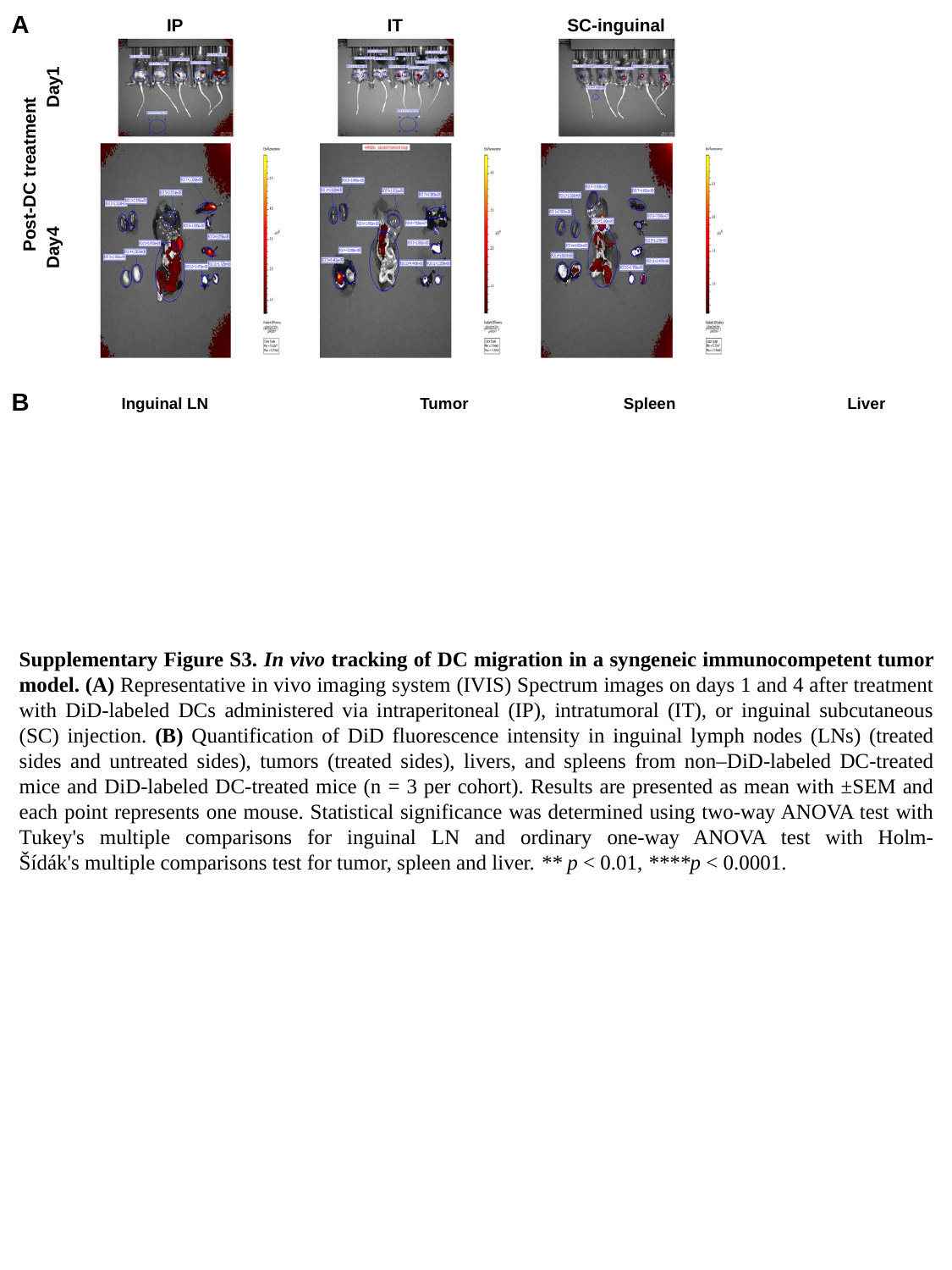

A
IP
IT
SC-inguinal
Day1
Post-DC treatment
Day4
B
Inguinal LN
Tumor
Spleen
Liver
Supplementary Figure S3. In vivo tracking of DC migration in a syngeneic immunocompetent tumor model. (A) Representative in vivo imaging system (IVIS) Spectrum images on days 1 and 4 after treatment with DiD-labeled DCs administered via intraperitoneal (IP), intratumoral (IT), or inguinal subcutaneous (SC) injection. (B) Quantification of DiD fluorescence intensity in inguinal lymph nodes (LNs) (treated sides and untreated sides), tumors (treated sides), livers, and spleens from non–DiD-labeled DC-treated mice and DiD-labeled DC-treated mice (n = 3 per cohort). Results are presented as mean with ±SEM and each point represents one mouse. Statistical significance was determined using two-way ANOVA test with Tukey's multiple comparisons for inguinal LN and ordinary one-way ANOVA test with Holm-Šídák's multiple comparisons test for tumor, spleen and liver. ** p < 0.01, ****p < 0.0001.
